# Searching for the best real-time RT-PCRs to detect Zika virus infections: the importance of comparing several protocols

**DOI:** 10.1590/1414-431X20187221

**Published:** 2018-05-21

**Authors:** F.M. de Moraes, D.L.A. Espósito, T.M. Klein, B.A.L. da Fonseca

**Affiliations:** 1 Departamento de Clínica Médica, Faculdade de Medicina de Ribeirão Preto, Universidade de São Paulo, Ribeirão Preto, SP, Brasil; 2 Departamento de Bioquímica e Imunologia, Faculdade de Medicina de Ribeirão Preto, Universidade de São Paulo, Ribeirão Preto, SP, Brasil

**Keywords:** Zika virus, Flavivirus, real time RT-PCR, Diagnosis

## Abstract

Clinical manifestations of Zika, dengue, and chikungunya virus infections are very similar, making it difficult to reach a diagnosis based only on clinical grounds. In addition, there is an intense cross-reactivity between antibodies directed to Zika virus and other flaviviruses, and an accurate Zika diagnosis is best achieved by real-time RT-PCR. However, some real-time RT-PCR show better performance than others. To reach the best possible Zika diagnosis, the analytic sensitivity of some probe-based real-time RT-PCR amplifying Zika virus RNA was evaluated in spiked and clinical samples. We evaluated primers and probes to detect Zika virus, which had been published before, and tested sensitivity using serum spiked and patient samples by real-time RT-PCR. When tested against spiked samples, the previously described primers showed different sensitivity, with very similar results when samples from patients (serum and urine) were analyzed. Real-time RT-PCR designed to amplify Zika virus NS1 showed the best analytical sensitivity for all samples.

## Introduction

Zika, a disease resulting from Zika virus (ZIKV) infection, has emerged recently and spread to several countries of the world at incredible speed. ZIKV is a positive-sense, single-stranded RNA virus that belongs to the *Flaviviridae* family, *Flavivirus* genus, and is transmitted primarily by *Aedes* mosquitos (1). Since its isolation, ZIKV has infected people in Africa, Asia, Pacific Islands, and more recently in Brazil, where large-scale spreading occurred. Zika symptoms are usually mild, characterized by low-grade fever, pruritic maculopapular rash, myalgia, arthralgia, conjunctivitis, and headache (2). Hospitalization due to severe clinical manifestation is uncommon, but the increasing number of cases of Guillain-Barré syndrome and congenital Zika virus syndrome occurring concomitantly to Zika outbreaks has created global concern around this disease (3).

In the New World, Zika was first identified in Brazil, where it was associated with an increased number of congenital Zika virus syndrome cases, but most of them lacked a definitive Zika diagnosis (4). Routine diagnosis of *Flavivirus* infections is usually made either through antibody detection by ELISA (enzyme-linked immunosorbent assay) or RT-PCR (reverse transcription polymerase chain reaction), depending on the phase of the disease. RT-PCR is used in the acute phase of diseases and IgM antibodies are usually detected in patients’ serum collected in the convalescence phase (3). The main problem with antibody detection is the cross-reactivity among the flaviviruses, making it difficult to differentiate these diseases, especially in areas where more than one *Flavivirus* circulate at the same time.

RT-PCR has been used to detect ZIKV RNA in many types of samples, including serum, plasma, urine, and saliva obtained during the acute phase (3,5). Although RT-PCR is a more expensive choice due to its equipment platforms and reagent costs, it is a rapid, sensitive, and specific method for ZIKV RNA detection and quantitation, but there are several protocols with different analytical sensitivities and, sometimes, it is difficult to choose the one that will be the best fit for the specific diagnosis of ZIKV infections (6). Due to possible sequence variation, a necessary step to solve this dilemma is the evaluation of RT-PCR protocols able to detect the viral strain circulating in a specific region. Finally, due to the Guillain-Barré Syndrome and congenital Zika virus syndrome associated to ZIKV infection, the correct diagnosis of this disease is very important. Here, we tested some probe-based real-time RT-PCRs used to detect ZIKV genome in clinical cases, evaluating the sensitivity and specificity of each protocol, aiming at a definition of which one would yield the most accurate diagnosis.

## Material and Methods

### ZIKV dilution

Ten-fold serial dilutions of ZikaSPH2015 strain (7) ranging from 106–100 copies/mL, titrated in Vero cells, were spiked in a serum sample from a healthy donor and were tested by real-time RT-PCR in duplicates. Samples were previously negative for the presence of all dengue serotypes by real-time RT-PCR and for dengue IgM and IgG antibodies by immunochromatographic test.

### Specificity test

To verify if all primers and probes are specific for ZIKV detection, different viruses were tested by real-time RT-PCR in triplicates. Dengue serotypes 1 to 4 (DENV-1 to 4) [DENV-1 (Hawaii strain), DENV-2 (New Guinea C strain), DENV-3 (H87 strain), and DENV-4 (H241 strain)], chikungunya (CHIKV) (BzH1 strain), yellow fever (YFV) (BeH111 strain), ZIKV Asian lineage (ZikaSPH2015 strain), and ZIKV African lineage (MR766 strain [ATCC VR-84]) were tested.

### Coinfection test

Zika, dengue, and chikungunya virus samples, titrated in Vero cells, were spiked in the same serum sample. A known quantity (104 virus) of each virus was combined as the following: ZIKV and DENV; ZIKV and CHIKV; ZIKV, DENV, and CHIKV, and only ZIKV as the positive control. All samples were tested by real-time RT-PCR in triplicate.

### Patient samples

Samples from ZIKV-infected patients were used with the patients’ permission and the study was approved by the Ethics Committee of Hospital das Clínicas-FMRP/USP (CEP-Comitê de Ética em Pesquisa Protocol No. 1.428.859).

### RNA extraction and real-time RT-PCR

Viral RNA was extracted with QIAamp Viral RNA Mini Kit (Qiagen¯, Germany), according to the manufacturer’s protocol and amplified by real-time RT-PCR, in a RealPlex 4 Thermocycler (Eppendorf¯, Germany), using TaqMan¯ Fast Virus 1-Step Master Mix kit (ThermoFisher¯, USA) and five different pairs of primers with their respective probe, referred throughout this article as ZIKV A to ZIKV E (Supplementary Table S1). The amount of each primer and probe used individually per reaction was 125 nM, 2.5 µL of master mix, 3 µL of RNA and the final volume was set to 10 µL. The cycling condition for all protocols was 10 min at 50°C to ensure cDNA synthesis, 1 minute at 95°C to activate DNA polymerase and inactivate the reverse transcriptase, and 45 cycles at 95°C for 15 s to denature cDNA and 60°C for 45 s to primer annealing and extension. The ten-fold serial dilution of plaque forming units (PFU) equivalents was used to test specificity and sensitivity of primer and probe sets. Table S1 shows each primer and probe sequence with its genome region and position, amplicon size, and their respective reference.

## Results

The real-time RT-PCR for specificity test have shown that all primers/probes can be used to detect ZIKV Asian lineage, while the African lineage could not be detected by ZIKV D. Two unspecific amplifications have occurred, but only one of the respective triplicates: YFV by ZIKV A and CHIKV by ZIKV D, what is probably due to contamination while pipetting the samples.

The cycle threshold (Ct) of each primer/probe set used in the real-time RT-PCR of the 10-fold serial dilution of ZIKV RNA is shown in Table 1. The sensitivity of each primer/probe is represented by the Ct, where the lower the value the higher the analytical sensitivity. Among all primers/probes, ZIKV D, designed by Pyke and coworkers (8), is the one that had the lowest Ct values followed by ZIKV E, while ZIKV C, designed by Tappe and coworkers (9), had the highest Cts, showing a very low analytical sensitivity. Besides, ZIKV C detected virus only until 103 copies/mL, while the other primers/probes could detect the virus in all dilutions.

**Table 1.** Cycle threshold of primers/probes protocols used for all Zika virus (ZIKV) RNA dilutions.

Virus/dilution	ZIKV A Ct	ZIKV B Ct	ZIKV C Ct	ZIKV D Ct	ZIKV E Ct
106	11.70±0.11	11.49±0.79	21.11±2.22	11.08±0.03	10.63±0.03
105	15.79±0.10	15.92±0.16	30.37±0.87	14.68±0.12	15.06±0.08
104	19.59±0.10	19.11±0.06	33.76±0.17	18.25±0.10	18.78±0.03
103	23.05±0.15	23.07±0.23	36.94±0.79	21.86±0.0	22.26±0.32
102	26.51±0.0	26.43±0.41		25.45±0.43	25.65±0.01
101	29.56±0.17	29.52±0.24		28.31±0.01	28.81±0.03
100	33.85±0.22	33.27±0.28		32.62±0.26	33.30±0.71

Data are reported as the mean of the duplicate values and standard deviations. Ct: cycle threshold.

The real-time RT-PCR performed with the coinfected samples (Table 2) have shown that there is no difference (two-way ANOVA test, P>0.05) between the Cts from positive control (ZIKV) and coinfected samples using any of those primers, which means that the presence of another virus, even from the same family and genus as ZIKV, does not have an effect on the sensitivity of primers and probes used in this study.

**Table 2.** Cycle threshold of primers/probes protocols used to test the presence of Zika virus (ZIKV) in coinfected samples.

	ZIKV	ZIKV+DENV	ZIKV+CHIKV	ZIKV+DENV+CHIKV
ZIKV A Ct	19.91±0.29	19.69±0.35	19.94±0.10	19.57±0.34
ZIKV B Ct	20.16±0.15	19.89±0.12	20.09±0.12	19.84±0.14
ZIKV C Ct	34.69±0.44	34.87±0.39	34.65±0.32	35.43±0.83
ZIKV D Ct	18.50±0.19	18.24±0.15	18.60±0.15	18.29±0.35
ZIKV E Ct	18.92±0.00	18.84±0.11	18.85±0.15	18.22±0.01

Data are reported as the mean of the triplicate values and standard deviations. Ct: cycle threshold; DENV: dengue; CHIKV: chikungunya.

The same real-time RT-PCR was performed with 17 clinical samples, 11 serum samples (S), and 6 urine samples (U), from clinically diagnosed Zika patients who previously tested negative for the presence of dengue viruses RNA by real-time RT-PCR. Compared to all primer/probe sets, ZIKV D and E were the most sensitive primers/probe sets for serum samples from patients 1 to 11. Comparing ZIKV D and E sensitivities, ZIKV D showed the best sensitivity detecting ZIKV in all samples while samples 10 and 11 were negative with ZIKV E. ZIKV C had the lowest sensitivity, detecting ZIKV RNA in only two samples and with high Cts. Again, in urine samples 12 to 17, ZIKV D and ZIKV C had the best and worst sensitivities, respectively (Table 3).

**Table 3.** Cycle threshold of primer/probe protocols used to test the presence of Zika virus (ZIKV) in serum (S) and urine (U) samples from infected patients.

Sample	ZIKV A Ct	ZIKV B Ct	ZIKV C Ct	ZIKV D Ct	ZIKV E Ct
1S	34.75	34.38		33.00	34.58
2S	32.48	32.96		31.18	32.44
3S	28.16	27.94	29.78	26.66	27.54
4S	26.03	25.48	37.66	24.69	26.22
5S	28.37	27.63		26.29	27.60
6S	32.71	32.74		31.57	32.47
7S				36.85	36.34
8S	37.43			35.31	37.35
9S	37.43	36.57		35.04	34.82
10S		37.62		35.95	
11S				36.77	
12U	36.84	35.97	39.56	33.20	35.62
13U	35.20	39.16		33.95	34.13
14U	30.67	31.29		29.74	29.55
15U	32.09	32.52		29.93	30.78
16U	33.80	34.70		31.73	33.96
17U	37.28	36.75		36.81	

Data are reported as cycle threshold (Ct).

It is known that the virus RNA is detectable in urine at a higher load and for a longer period of time than in serum samples (10,11). However, ZIKV C primers/probe could not detect ZIKV RNA in these samples, ZIKV E could not detect the virus in one of them while ZIKV A, B, and D primers/probe sets detected in all of them (Table 3).

## Discussion

Zika, dengue, and chikungunya are important arthropod-borne virus infections of worldwide concern, but the correct diagnosis of these diseases based solely on clinical grounds has been an enormous challenge due to the similarity between their clinical manifestations. Diagnosis based on the antibody detection is also problematic as the cross-reactivity between ZIKV and DENV antibodies poses an additional problem. Lanciotti and coworkers (12) reported that ZIKV-infected patients had IgM antibodies against dengue viruses, especially if ZIKV was a secondary flavivirus infection. Thus, in areas where other flavivirus infections are prevalent, such as in the Americas, it is expected an extensive cross-reactivity between dengue and Zika IgM antibodies, which could lead to the misdiagnosis of these diseases (12). Thus, serologic testing alone is not sufficient to confirm ZIKV infection, especially in patients with secondary flavivirus infections.

For a long time, virus isolation in cell cultures was considered the “gold standard” for virus detection. However, due to the lengthy incubation period needed for Zika isolation as well as the need for technical training and skills for cell manipulation, molecular methods are gaining space in diagnostic laboratories (13). Among these molecular techniques, real-time RT-PCR is the most sensitive, specific, and rapid method to detect and quantify genetic material due to the use of specific primers and probes specially designed for specific viruses (6). Effectiveness for diagnostic purposes is best when primers and probes are designed into a conserved region of a virus genome and have no homology with other viruses.

Lanciotti and coworkers (12) evaluated the sensitivity of primers ZIKV A and B by testing dilutions of known copy numbers of an RNA transcript and data showed that ZIKV B was more sensitive than ZIKV A, with a sensitivity of 25 and 100 copies/mL, respectively. These results differ from ours because both primers could detect until up to 1 copy/mL when tested with our standard curve. The reason for this difference is most probably due to the PFU titrated virus that we used as gold standard samples. When using virus isolated from cells as real-time RT-PCR positive controls, the genomes of non-infective viral particles are also amplified, artificially increasing the viral load.

Pyke and coworkers (8) developed two pairs of primers for RNA ZIKV detection based on ZIKV NS1 and E protein sequences, and our results showed that the NS1-based protocol (ZIKV D) amplified ZIKV RNA in a lower Ct than the E-based protocol (ZIKV E). ZIKV D primers/probes had also a better performance than all the other primers/probes, including those designed by Lanciotti et al (12). An explanation might be related to the strain that the authors used to design their primers/probes (ZIKV 2007 strain from Micronesia), which might be slightly different from the one currently circulating in Brazil. Since we used samples from Brazilian patients who have had autochthonous transmission, these results can be easily explained. However, ZIKV D is not suitable for African lineage detection since it does not anneal with this lineage. Corman and coworkers (14) published different results from ours, showing that the e-based Pyke (ZIKV e) assay had better analytical sensitivity than Pyke ns1 primers/probe, and both Lanciotti assays presented better ZIKV detection than Pyke’s. A possible explanation for these discordant results is that Corman used a different strain from ours to test the efficacy of primers. Each strain has some mismatches in primer binding site that could increase or decrease the ability of annealing and amplification of ZIKV by these primers, possibly giving rise to discordant sensitivity results between these two experiments. Besides, we conducted our experiments with different protocols and kits (RNA extraction and real time RT-PCR kits) that were used by Corman, probably contributing with these different results in the sensitivity of the tested primers/probe sets.

Tappe and coworkers (9) designed ZIKV C primers to diagnose ZIKV infection of a German patient returning from a vacation in Thailand. However, the virus could not be detected in serum and the diagnosis was confirmed by serological tests. Besides the lower analytical sensitivity observed with ZIKV C in our study, the real-time RT-PCR negative result observed in their case report might be due to the day the sample was collected (about 10 days after symptoms onset), when the viral load might be undetectable. Nevertheless, Lanciotti and coworkers (12) showed in their work that a sample collected after 11 days of beginning of symptoms was positive when using either ZIKV A or B primers, which proves their higher sensitivity compared to ZIKV C. The possible presence of mismatches between ZIKV C primers/probes and ZikaSPH2015 sequences could be the reason for the lower performance of these primers to detect ZIKV RNA in our samples, evidencing the need of a careful analysis of the available real-time RT-PCR protocols in order to obtain the most reliable results, especially with RNA viruses where the mutation rate is highest than with DNA viruses.

The sequence used by Tappe and coworkers (9) had several differences in its composition compared to the Brazilian strain, resulting in a low sensitivity of ZIKV C primers/probes to detect ZIKV infections in Brazilian patients. Actually, there were 7 mismatches between ZikaSPH2015 sequence and the regions used by the authors to design ZIKV C primers/probes. On the other hand, the ZikaSPH2015 sequence has a full match with the NS1-based primers/probes used by Pyke et al (8). These facts might explain the higher sensitivity when using ZIKV D to amplify ZIKV RNA from Brazilian samples compared with the lower sensitivity obtained with ZIKV C primers/probes used to amplify our samples.

In conclusion, the spread of ZIKV around the world shows the need for a rapid, specific, and sensitive diagnostic method. Real-time RT-PCR fulfills all of these requirements since it is possible to design protocols presenting no cross-reactivity with other flaviviruses and quantify virus particles and the result is obtained in a matter of hours. In our study, we evaluated different protocols using primers and probes described in the literature aiming at the most accurate diagnosis of human ZIKV infections. After testing these protocols in variable ZIKV RNA concentrations and in samples of the acute phase of the disease, as a whole ZIKV D showed the best sensitivity for the detection of the lineage currently circulating worldwide. In addition, this study showed the importance of designing primers and probes based on the strain/lineage of the virus circulating in a specific area in order to obtain the best diagnostic results.

## Supplementary Material

Click here to view [pdf]
